# MicroRNA-146a promotes proliferation, migration, and invasion of HepG2 via regulating FLAP

**DOI:** 10.1186/s12935-022-02568-0

**Published:** 2022-04-11

**Authors:** Huihui Wang, Shubing Zhang, Tao Li, Lianzi Wang, Wei Lv, Shanshan Wang, Dongyue Ma, Yan Zang, Xinyue Zhu, Yuanhong Xu, Lan Zheng, Jilong Shen, Wei Wei

**Affiliations:** 1 Department of Clinical Laboratory, The Third People’s Hospital of Bengbu, Bengbu, 233000 Anhui China; 2 Department of Clinical Laboratory, the First Affiliated Hospital of Anhui Medical University, Shushan District, No. 218 Jixi Road, Hefei, 230032 Anhui China; 3 Institute of Clinical Pharmacology, Key Laboratory of Anti-Inflammatory and Immune Medicine, Ministry of Education, Anhui Collaborative Innovation Center of Anti-Inflammatory and Immune Medicine, Anhui Anti-Inflammatory and Immune Medicine Innovation Team, Anhui Medical University, Hefei, 230032 China; 4 The Key Laboratory of Microbioloy and Parasitology of Anhui Province, The Provincial Key Laboratory of Zoonoses of High Institutions in Anhui, Hefei, 230032 China

**Keywords:** MircoRNA-146a, 5-Lipoxygenase Activating Protein (FLAP), Hepatocellular carcinoma (HCC)

## Abstract

Abnormal expression of 5-Lipoxygenase Activating Protein (FLAP) has been detected in many tumor cells. MicroRNAs (miRNAs) negatively regulate gene expression post-transcriptionally by binding to the 3'–untranslated region (3'–UTR) of the target mRNA sequences and have been shown to be involved in various types of cancers. Herein, we aimed to demonstrate the expression of miR-146a and FLAP in human HCC tissues and liver cancer cell lines. We demonstrated that miR-146a expression is overexpressed, while FLAP protein and mRNA are suppressed in hepatocellular carcinoma tissues and HepG2 cells compared to para-carcinoma tissues and HL–7702 cells. Dual luciferase reporter gene assay showed that miR-146a-5p can directly target FLAP mRNA. Knockdown of miR-146a also resulted in increased FLAP expression of cancer cells. Additionally, miR-146a silencing or restoration of FLAP led to a reduction of HepG2 cell proliferation, cell cycle progression, migration, and invasion. This study showed that miR-146a has a stimulatory role in HepG2 cells and promotes HepG2 cell migration and invasion by targeting FLAP mRNA. Thus, miR-146a may be a tumor promoter and a potential therapeutic target for the treatment of HCC patients.

## Introduction

Liver cancer, the sixth most diagnosed cancer and the fourth leading cause of cancer death worldwide, leads to an estimated 841,000 new cases and 782,000 deaths annually [1]. Hepatocellular carcinoma (HCC), the most common primary liver cancer, is the second leading cause of cancer-associated death in the world [2]. The progress of HCC is complex and multifactorial caused by pathogenic factors such as chronic infection with Hepatitis B virus (HBV) or Hepatitis C virus (HCV), liver cirrhosis, habitual alcohol abuse, and aflatoxin B1 exposure [3]. Currently, surgical resection, liver transplantation, liver directed therapy, and systemic therapy are major treatment options of HCC. However, only 15% of patients are eligible for curative treatments including surgical resection and liver transplantation since most patients develop symptoms at an advanced disease stage [4]. Recently, cancer immunotherapy has emerged as one of the most promising approaches for cancer treatment. The mainstay of treatment for advanced hepatocellular carcinoma (HCC) was the tyrosine kinase inhibitor (TKI) sorafenib [5]. Atezolizumab and bevacizumab in combination are now considered standard care in the first-line setting in patients with advanced HCC [6]. Novel drug delivery systems such as nanoparticles or liposomes substantially control the severity of HCC and they can be considered as a promising nanocarrier in the management of HCC [7, 8].

MicroRNAs (miRNAs), with the length of 18–22 nucleotides, have been shown to downregulate target mRNA expression post-transcriptionally by binding to the 3'–untranslated region (UTR) of the target mRNA sequences [9]. Recently, microRNA-146a (miR-146a) was reported to be involved in a variety of pathogenic pathways associated with hepatocarcinogenesis, including HBV and HCV infections [3, 10]. MiR-146a has also been shown to promote the angiogenic activity of endothelial cells in HCC patients [11]. However, some studies have found that methylated miR-146a promoter leads to a significant decrease of its expression in HCC [12]. Furthermore, activated miR-146a inhibited cancer migration, invasion, and metastasis by downregulating vascular endothelial growth factor through dual pathways in HCC. MiR-146a exhibits different expression patterns, which regulate various characteristics and cellular processes, particularly during an inflammatory state of HCC, by downregulating various target genes [13–15].

The metabolic pathway of arachidonic acid (AA) has two main branches: 1) AA is converted by COX-1 and COX-2 into prostaglandins (PG) and thromboxane (TX), and 2) the actions of 5-lipoxygenase (5-LO) and 5-Lipoxygenase Activating Protein (FLAP) create leukotrienes (LT) in the other arm of the pathway [16]. 5-LO and FLAP were mainly expressed in a variety of myeloid cells, including neutrophils, eosinophils, monocytes/macrophages, dendritic cells, mast cells, and B lymphocytes [17]. Abnormal expression of 5-LO and FLAP was detected in many tumor cells, in comparison to normal expression in healthy white blood cells [18–20]. FLAP is primarily located in the nuclear membrane and endoplasmic reticulum of leukocytes in peripheral blood as observed through immunoelectron microscopy [21]. Previous study has shown that FLAP is universally expressed in 20 types of epithelial cancer cell lines including colon cancer, lung cancer, breast cancer, prostate cancer [22]. Overexpressed FLAP was associated with a reduced survival rate in human breast cancer; furthermore, inhibiting FLAP activity weakens the growth of breast tumor cells [23]. However, the expression and role of FLAP in HCC is unknown. Hypoxia induces FLAP expression in endothelial cells by enhancing the binding of HIF-1α and NF-κB to the FLAP promoter, whereas miR-135a and miR-199a-5p target the 3'–UTR of FLAP mRNA and down-regulate the FLAP gene expression [24], suggesting that FLAP expression can be regulated through transcriptional activation and post-transcriptional regulation. Interestingly, the FLAP 3'–UTR contains a highly conserved miR-146a binding site [25]. We postulated that perhaps miR-146a might regulate FLAP, a key activating protein of the AA metabolic pathway, in HCC.

The expression of miR-146a may serve as a prognostic biomarker and a potential therapeutic target for the treatment of HCC patients. This study aimed to demonstrate the expression of miR-146a and FLAP in human HCC tissues and liver cancer cell lines. Furthermore, we theorized that FLAP is a target of miR-146a, since the presence of binding sites between the 3'–UTR of FLAP and miR-146a-5p was predicted by TargetScan software. It is interesting to discover the role of miR-146a and FLAP in HCC.

## Materials and methods

### Study approval and sample collection

The study volunteers provided written informed consent in accordance with the ethical standards of the institutional and national research committee. This study met the standards of the Declaration of Helsinki of 1964 and its later amendments. The Ethics Committee of the First Affiliated Hospital of Anhui Medical University approved this study (PJ2021-02-34). Sixteen human HCC tissues and para-carcinoma tissues were collected from the First Affiliated Hospital of Anhui Medical University.

### Cell culture

The HCC cell lines HepG2, Hep3B, and PLC (Wuhan Cell Bank, Chinese Academy of Sciences) were maintained in Dulbecco’s Modified Eagle’s Medium (DMEM) (HyClone Laboratories, Logan, Utah, USA). Human normal liver cell line, HL-7702 (Wuhan Cell Bank, Chinese Academy of Sciences), were maintained in an RPMI1640 medium (HyClone Laboratories). All the media were supplemented with 10% fetal bovine serum (FBS, WISENT, Canada) and 1% Penicillin/Streptomycin, and all the cells were incubated at 37 °C in 5% CO2 incubator. Mycoplasma testing of cells was regularly performed using the Mycoplasma Detection kit (Solarbio, Beijing, China).

### PCR

Total RNA was isolated using TRIzol reagent (InvitrogenTM, Waltham, Massachusetts, USA). Reverse transcriptase (RT)-PCR: cDNA was generated from 500 ng of RNA per sample using M-MLV Reverse Transcriptase (Accurate Biotechnology, Hunan). Realtime PCR (qRT-PCR) was performed by SYBR® Green Premix Pro Taq HS qPCR Kit (Accurate Biotechnology, Hunan) at 95 °C for 30 and 15 s, and at Tm-2 °C for 30 s, at 75 °C for 15 s for a total of 40 cycles. For FLAP, the forward primer sequence was TCCTCGCTGTGCTCTGGTCTG, and the reverse primer sequence was AGGGGTGCTCTGCGTTCTCTC. Glyceraldehyde 3-phosphate dehydrogenase (GAPDH) forward primer sequence was CAGCCTCAAGATCATCAGCA and reverse primer was AGGGGTGCTCTGCGTTCTCTC. MiRNA was isolated using miRCute miRNA Extraction Isolation Kit (Tiangen, Sichuan, China). RT-PCR: miRNA cDNA was generated using miRCute Plus First-strand miRNA cDNA Synthesis Kit (Tiangen). miR-146a expression was detected with miRCute Plus MicroRNA Fluorescence Quantitative Assay Kit (SYBR Green®) (Tiangen) at 95 ℃ for 15 min for one cycle, and 95 °C for 20 s, 60 °C for 34 s, 75 °C for 15 s, 60 °C for 34 s for 40–45 cycles. For miR-146a, the forward primer sequence was GCGCGTGAGAACTGAATTCCATGGGT, and the reverse primer was provided by miRCute Plus MicroRNA Fluorescence Quantitative Assay Kit (SYBR Green®); the forward and reverse primer sequences of U6 were provided by the assay kit.

### Western blot

Antibodies were used against FLAP (1:1000; Abcam, Cambridge, UK), Cyclin D1 (1:1000; Proteintech, Illinoise, USA), Cyclin B1 (1:1000), Cyclin E1 (1:1000), CDK2 (1:1000), CDK4 (1:1000), P21(1:1000), and Vimentin (1:2000). Proteins were detected using a gel documentation system (ChemiDocTM XRS + System, Bio-Rad Laboratories, Hercules, Canada).

### Luciferase assays

293 T cells were seeded in a 96-well plate format at a density of 1 $\times$ 10^3^ cells/well. Transfection was carried out when the cell density reached 50%. 293 T cells were transfected with 0.16 µg luciferase construct (h-FLAP-wild-type 3'–UTR target plasmid, h-FLAP-mutated 3'–UTR target plasmid) and 5 pmol/µL of hsa-miR-146a-5p/Negative Control (NC) using LipofectamineTM 3000 (InvitrogenTM) according to the manufacturer’s suggested protocol. 6 h later, the medium was replaced with DMEM with 10% FBS, and 48 h after transfection, Firefly luciferase activity (internal parameter) and Renilla luciferase activity (luminescence) was measured using the Renilla-Glo® Luciferase Assay System (Promega, Madison, Wisconsin, USA) according to the manufacturer’s suggested protocol. 96 wells were placed in the enzyme plate analyzer to detect the glow-type signal of Renilla luciferase.

### Lentivirus infection

HepG2 cells were seeded in a 12-well plate format at a density of 1 $\times$ 10^5^ cells/well. According to the multiplicity of infection (MOI value), the corresponding volume of viral fluid (HBLV-h-FLAP-ZsGreen-PURO, HBLV-ZsGreen-PURO, HBLV-has-mir-146a-ZsGreen-PURO, HBLV-has-mir-146a-sponge-ZsGreen-PURO, HBLV-ZsGreen-PURO NC) was calculated and added into the cells. 4 h after virus infection, 500 µL of DMEM was added to replenish the culture volume. 24 h after infection, the culture medium containing the virus was replaced with DMEM with 10% FBS. The expression efficiency of GFP was observed by fluorescence microscopy 72 h after infection. DMEM with 10% FBS and 2 µg/mL Puromycin was used to screen cell lines with stable transduction. Flow cytometry was used to detect the transfection efficiency of stably transfected cell lines, and if the transfection efficiency was more than 90%, it could be used for subsequent functional experiments.

### FLAP-siRNA knock-down

HepG2 cells were seeded in a 6-well plate format at a density of 2 $\times$ 10^5^ cells/well and transfected with 20 nM of siRNA against FLAP or non-targeting control siRNA (GenePharma, Shanghai, China) using Lipofectamine^TM^ RNAiMAX Reagent (Invitrogen^TM^). 6 h after the transfection, the cells were switched to DMEM with 10% FBS. Verification of RelB knockdown was done by qRT-PCR within 24 h after transfection, or within 48 h after transfection with Western blot (WB).

### CCK8 assays

HepG2 cells were seeded in a 96-well plate format at a density of 1 $\times$ 10^3^ cells/well. After incubation at 37 °C for 24 h, 10 µL CCK8 solution (BestBio, Washington, USA) was added to each well according to the manufacturer’s suggested protocol. Optical Density (OD) value at 450 nm was determined with a microplate analyzer (Elx × 808, BioTek, Vermont, USA).

### Apoptosis assays

HepG2 cells were seeded in a 6-well plate format at a density of 2 $\times$ 10^5^ cells/well. Cells were collected when the cell density reached 70%. The cells were suspended with 100 µL 1 × Annexin V Apoptosis (APC) (BestBio) binding solution at a concentration of 1–5 $\times$ 10^6^ cells/mL. Annexin V-APC and propidium iodide (PI) solution (BestBio) was added according to the manufacturer’s suggested protocol. Flow cytometry (FC500, Beckman, California, USA) was used to detect cell apoptosis.

### Wound-healing scratch assays

HepG2 cells were seeded in a 6-well plate format at a density of 2 $\times$ 10^5^ cells/well. With the help of a sterile ruler tool, a sample gun was used to draw a line in the 6-well plate at a vertical and uniform speed. The width of cell scratch at 0 h was observed and photographed as a control. After incubation at 37 °C for 48 h, the width of cell scratch at 48 h was observed and photographed.

### Transwell migration assays

10 mg/mL Matrigel was placed in a refrigerator at 4 ℃ and melted overnight, and then diluted six times with DMEM. 60 µL of diluted Matrigel was added to the upper chamber of Transwell (0.8 µm) 24-well plate. After incubation at 37 °C for 2 h, the Matrigel was solidified. HepG2 cells were seeded in the upper chamber of Transwell 24-well plate at a density of 0.5 $\times$ 10^5^ cells/well using serum-free high glucose DMEM. 500 µL of DMEM with 20% FBS was added to the lower chamber of the Transwell 24-well plate. 24 h later, the chambers were stained with 0.1% crystal violet for 30 min.

### Statistics

A two-tailed Student’s *t*-test was used to determine statistical significance. A P-value of P < 0.05 was determines as statistically significant. One-way analysis of variance (ANOVA) was used for multiple group comparisons. GraphPad Prism 6.0 software was used for statistical analysis of experimental data. Asterisks represent the level of significance: *P = 0.05, **P = 0.01, ***P = 0.001, and ****P = 0.0001. All experiments were repeated three times.

## Results

### MiR-146a expression is elevated and FLAP expression is reduced in hepatocellular carcinoma

**Fig. 1:**
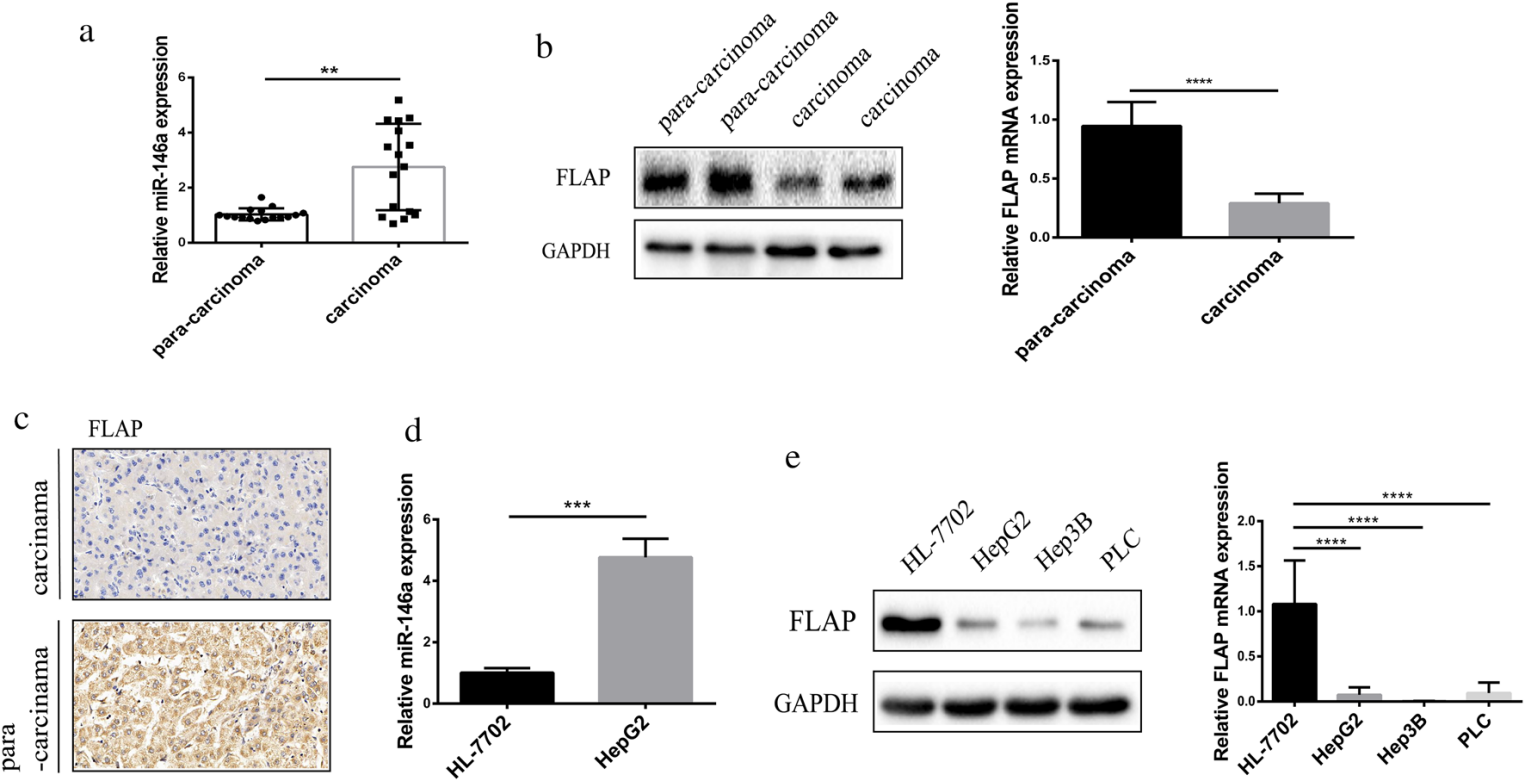
MiR-146a is overexpressed and FLAP is less expression in hepatocellular carcinoma. **a** miR-146a transcript levels in human hepatocellular carcinoma tissues and para-carcinoma tissues. **b** FLAP protein and transcript levels in human hepatocellular carcinoma tissues and para-carcinoma tissues. **c** Representative images (× 40) of immunohistochemical FLAP staining (brown) of hepatocellular carcinoma tissues and para-carcinoma tissues. **d** miR-146a transcript levels in liver cancer cell lines HepG2, Hep3B, PLC and normal liver cell line HL-7702. **e** FLAP protein and transcript levels in liver cancer cell lines HepG2, Hep3B, PLC and normal liver cell line HL-7702. Data are presented as mean ± SEM. **P < 0.01, ****P < 0.0001

To investigate whether miR-146a was involved in HCC, we first examined its expression in 16 human HCC tissues and para-carcinoma tissues. MiR-146a expression was higher in HCC tissues than in para-carcinoma tissues (Fig. 1a). Importantly, there were no differences in miR-146a expression between HCC tissues and para-carcinoma tissues in six cases (37.5%). We also found that FLAP expression was lower in HCC tissues than in para-carcinoma tissues (Fig. 1b, c). Next, we compared miR-146a and FLAP mRNA and protein expression in human normal liver cell line, HL-7702 cells (our experimental control), and three liver cancer cell lines: HepG2, Hep3B, and PLC cells. Consistent with the tissue results, we found that miR-146a expression was higher in HepG2 cells than in HL-7702 cells (Fig. 1d). Additionally, FLAP protein and mRNA were reduced in HepG2, Hep3B, and PLC cells than those in control cells (Fig. 1e).

### MiR-146a-5p directly targets FLAP mRNA

**Fig. 2:**
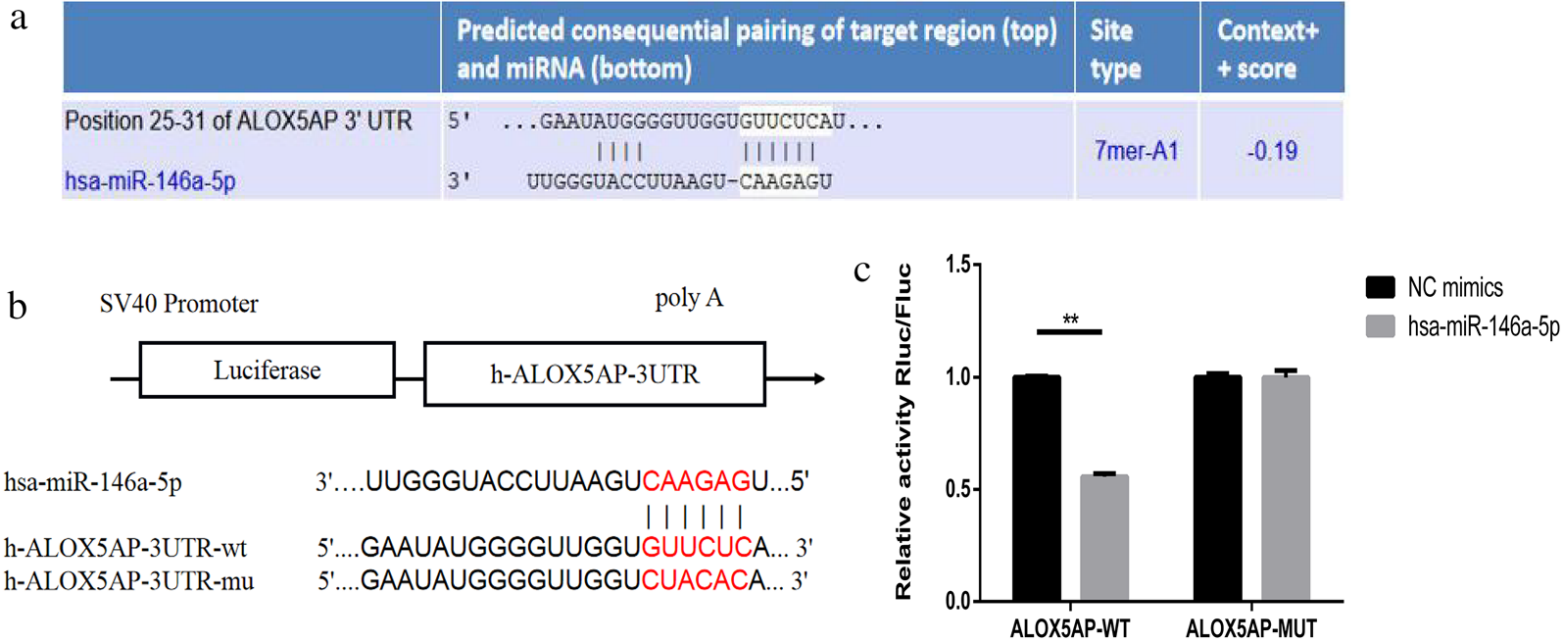
MiR-146a-5p directly target FLAP mRNA. **a** Target genes of miR-146a-5p were predicted by TargetScan. **b** The construction of a 3'UTR WT and MUT plasmid containing FLAP for dual luciferase reporter gene assay. **c** Dual-luciferase activity assay confirmed the interaction between miR-146a-5p and ALOX5AP. Data are presented as mean ± SEM. **P < 0.01

The presence of binding sites between the 3'–UTR of FLAP and miR-146a-5p was predicted by TargetScan software (Fig. 2a). Furthermore, qRT-PCR and WB detection showed that the expression level of FLAP was downregulated in HCC tissues and HepG2 cells (Fig. 1b, c, e), which was contrary to the expression trend of miR-146a-5p in HCC tissues and HepG2 cells (Fig. 1a, d). To further verify the structural similarities between FLAP and miR-146a-5p, we constructed a 3'–UTR wild-type (WT) and mutated (MUT) plasmid containing FLAP for dual luciferase reporter gene assay (Fig. 2b). The results of the dual luciferase reporter gene assay showed that, compared to normal control (NC), mimic, miR-146a-5p mimic could significantly down-regulate the luciferase activity of h-ALOX5AP-3'–UTR-WT. Furthermore, miR-146a-5p mimic had no significant effect on the luciferase activity of h-ALOX5AP-3'–UTR-MUT (Fig. 2c). Together, these results demonstrate that miR-146a-5p can directly target FLAP mRNA.

### MiR-146a promoted HepG2 cell proliferation, cycle progression, migration, and invasion

**Fig. 3:**
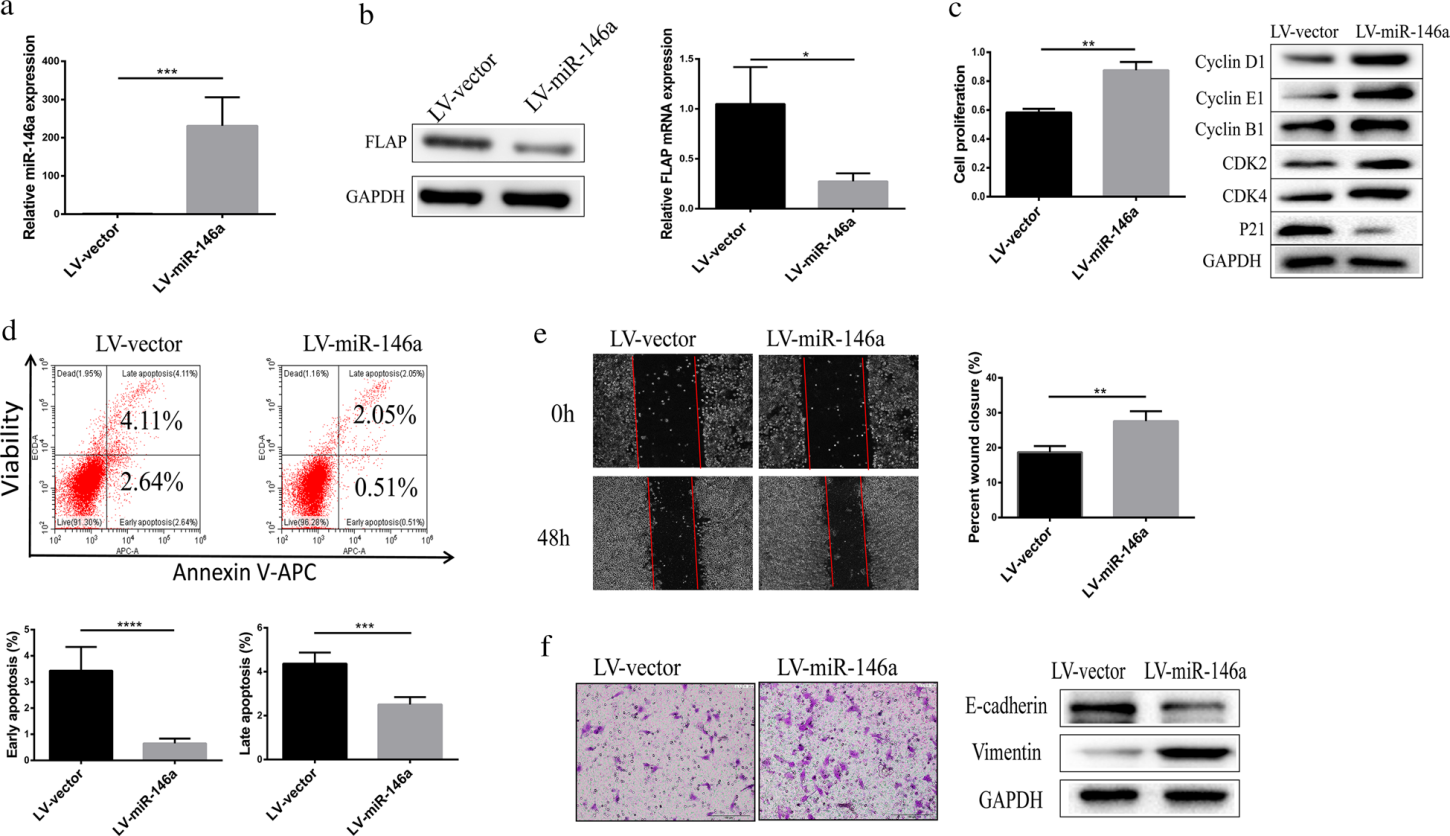
MiR-146a promoted HepG2 cell proliferation, cycle progression, migration and invasion. **a** miR-146a transcript levels of HepG2 cells transfected with control (LV-vector) or Lentivirus packaged miR-146a overexpression vector (LV-miR-146a). **b** FLAP protein and transcript levels of HepG2 cells transfected with control (LV-vector) or Lentivirus packaged miR-146a overexpression vector (LV-miR-146a). **c** Representative CCK-8 proliferation assays (left) and the cell cycle regulatory proteins expression in HepG2 cells. **d** Flow cytometry analysis (left) and quantitation (right) in HepG2 cells. **e** Representative wound-healing scratch assays (left) and quantitation (right) in HepG2 cells. **f** Representative matrigel invasion assay (left) and adhesion markers expression (right) in HepG2 cells. Data are presented as mean ± SEM. n = 3; **P < 0.01, ***P < 0.001,****P < 0.0001

Given that miR-146a expression was elevated in HCC, we speculated whether miR-146a promotes HCC cell proliferation. To investigate this, HepG2 cells were stably transfected with control (LV-vector) or Lentivirus packaged miR-146a overexpression vector (LV-miR-146a). QRT-PCR (Fig. 3a) confirmed that the LV-miR-146a substantially elevated miR-146a expression. To investigate the effect of increased miR-146a on FLAP expression, we found that the LV-miR-146a substantially reduced FLAP protein and mRNA expression (Fig. 3b). We followed proliferation for 24 h with CCK8 assays and found that LV-miR-146a significantly increased the proliferation of HepG2 cells (Fig. 3c).

Next, the expression of the cell cycle regulatory proteins was examined: CDK4 (G1 phase), CDK2 (G1 phase), Cyclin E1 (S-phase transition), Cyclin B1 (transition from G2 to M), Cyclin D1 (throughout the cell cycle), p21 (cell cycle inhibitor) [26]. Expression of Cyclins D1, E1, B1, CDK2 and CDK4 was efficiently increased in cells with miR-146a overexpression, whereas the expression of p21 was reduced (Fig. 3c). These results suggest that high miR-146a expression promotes HepG2 cell proliferation by inducing G2/M cell cycle arrest.

Next, we evaluated the effect of LV-miR-146a on cell apoptosis. Flow cytometry analysis revealed that, LV-miR-146a significantly decreased the apoptosis of HepG2 cells (Fig. 3d).

Metastasis, the main cause of most cancer-related deaths, occurs after cells undergo the epithelial-to-mesenchymal transformation (EMT), which is an important process of tumor invasion and early metastasis [27, 28]. The wound-healing scratch assays simulates the process of cell migration in vivo and is suitable for studying cell migration caused by the interaction between cells and the extracellular matrix. To determine whether miR-146a was required for tumor cell migration, we performed wound-healing scratch assays in cells in which miR-146a was overexpressed. At 48 h post-scratch, HepG2 cells expressing high miR-146a were significantly more migratory than LV-vector (Fig. 3e). To evaluate whether miR-146a was required for tumor cell invasion, we performed Matrigel invasion assay in cells with miR-146a overexpression. Compared to LV-vector, HepG2 cells expressing high miR-146a were significantly more capable to invade through Matrigel (Fig. 3f). Next, we examined the expression of adhesion markers: E-cadherin and vimentin associated with EMT. We found that cells in which miR-146a was overexpressed had lower E-cadherin expression and higher vimentin expression than in LV-vector (Fig. 3f). Together, these results suggest that miR-146a promotes migration and invasion behaviors of HepG2 cells.

### Silencing of miR-146a reduced HepG2 cell proliferation, cycle progression, migration, and invasion

**Fig. 4:**
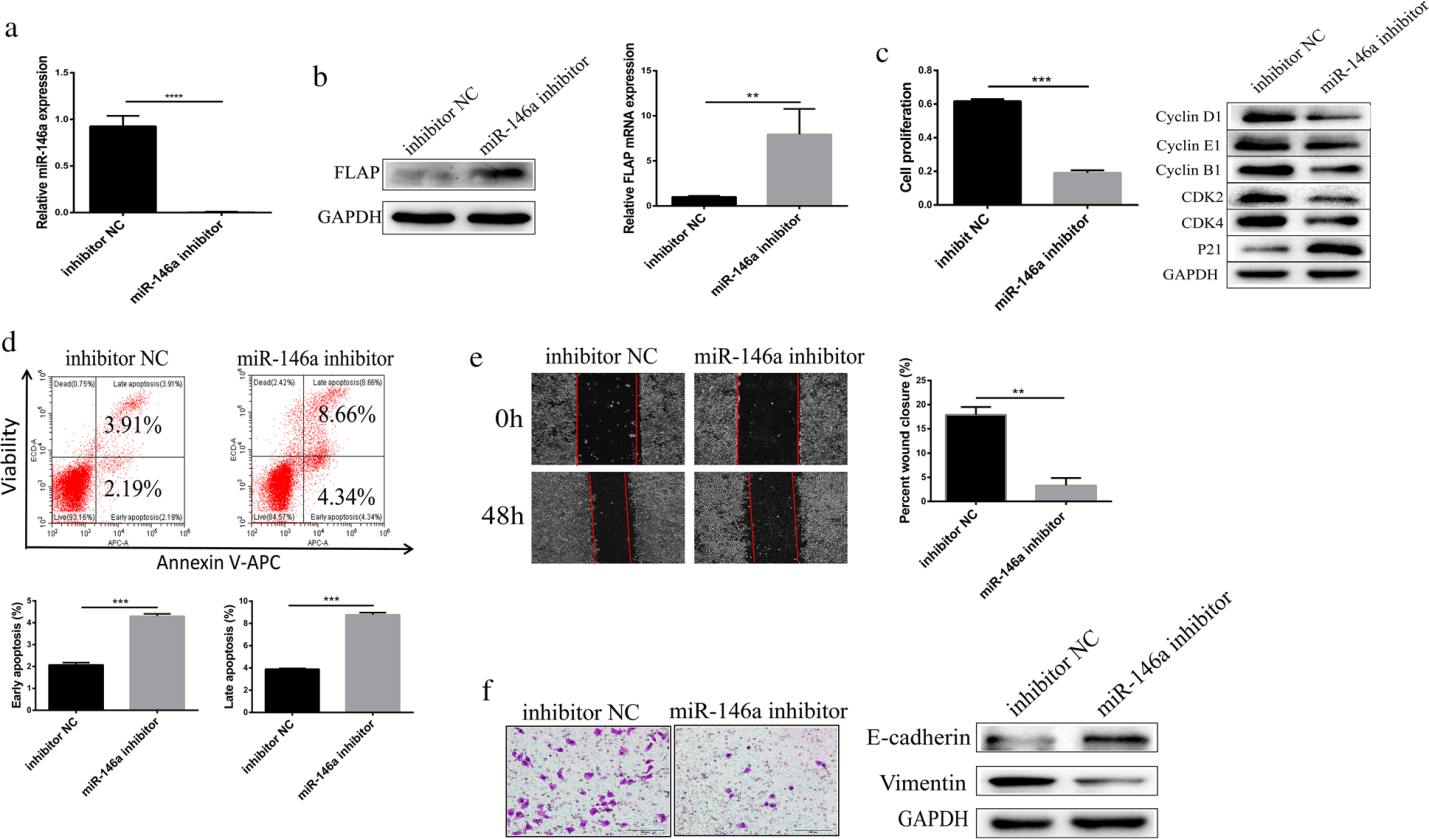
MiR-146a silencing reduced HepG2 cell proliferation, cycle progression, migration and invasion.** a** miR-146a transcript levels of HepG2 cells transfected with control (inhibitor NC) or Lentivirus packaged miR-146a knockdown vector (miR-146a inhibitor). **b** FLAP protein and transcript levels of HepG2 cells transfected with control (inhibitor NC) or Lentivirus packaged miR-146a knockdown vector (miR-146a inhibitor). **c** Representative CCK-8 proliferation assays (left) and the cell cycle regulatory proteins expression in HepG2 cells. **d** Flow cytometry analysis (left) and quantitation (right) in HepG2 cells. **e** Representative wound-healing scratch assays (left) and quantitation (right) in HepG2 cells. **f** Representative matrigel invasion assay (left) and adhesion markers expression (right) in HepG2 cells. Data are presented as mean ± SEM. n = 3; *P < 0.05, **P < 0.01, ***P < 0.001

We stably transfected HepG2 cells with control (inhibitor NC) or Lentivirus packaged miR-146a knockdown vector (miR-146a inhibitor), to further prove that miR-146a silencing reduced HepG2 cell proliferation. To investigate this, qRT-PCR (Fig. 4a) confirmed that the miR-146a inhibitor substantially knocked down miR-146a expression. Consistent with the results in Fig. 3b, we found that the miR-146a inhibitor substantially increased FLAP protein and mRNA expression (Fig. 4b). We followed proliferation for 24 h with CCK8 assays and found that miR-146a inhibitor significantly reduced the proliferation of HepG2 cells (Fig. 4c).

Next, we examined the expression of the cell cycle regulatory proteins: Cyclin D1, Cyclin E1, Cyclin B1, p21, CDK4, and CDK2. Expression of Cyclins D1, E1, B1, CDK2, and CDK4 was efficiently decreased in cells in which miR-146a was knocked down, whereas the expression of p21 was elevated (Fig. 4c). These results suggest that miR-146a silencing reduced HepG2 cell proliferation by inducing G2/M cell cycle arrest.

Next, we evaluated the effect of miR-146a inhibitor on cell apoptosis. Flow cytometry analysis revealed that, miR-146a inhibitor significantly increased the apoptosis of HepG2 cells (Fig. 4d).

To determine whether miR-146a silencing reduced HepG2 cell migration, we performed wound-healing scratch assays in cells with miR-146a knockdown. At 48 h post-scratch, HepG2 cells with miR-146a silencing were significantly less migratory than inhibitor NC (Fig. 4e). To evaluate whether miR-146a silencing reduced HepG2 cell invasion, we performed Matrigel invasion assay in cells in which miR-146a was knocked down. Compared to inhibitor NC, HepG2 cells with miR-146a silencing were significantly less capable to invade through Matrigel (Fig. 4f). Next, we examined the expression of adhesion markers: E-cadherin and vimentin associated with EMT. We found that cells in which miR-146a was knocked down had higher E-cadherin expression and lower vimentin expression than inhibitor NC (Fig. 4f). These results suggest that miR-146a silencing reduced HepG2 cells migration and invasion.

### High FLAP expression inhibited HepG2 cell proliferation, cycle progression, migration, and invasion

**Fig. 5:**
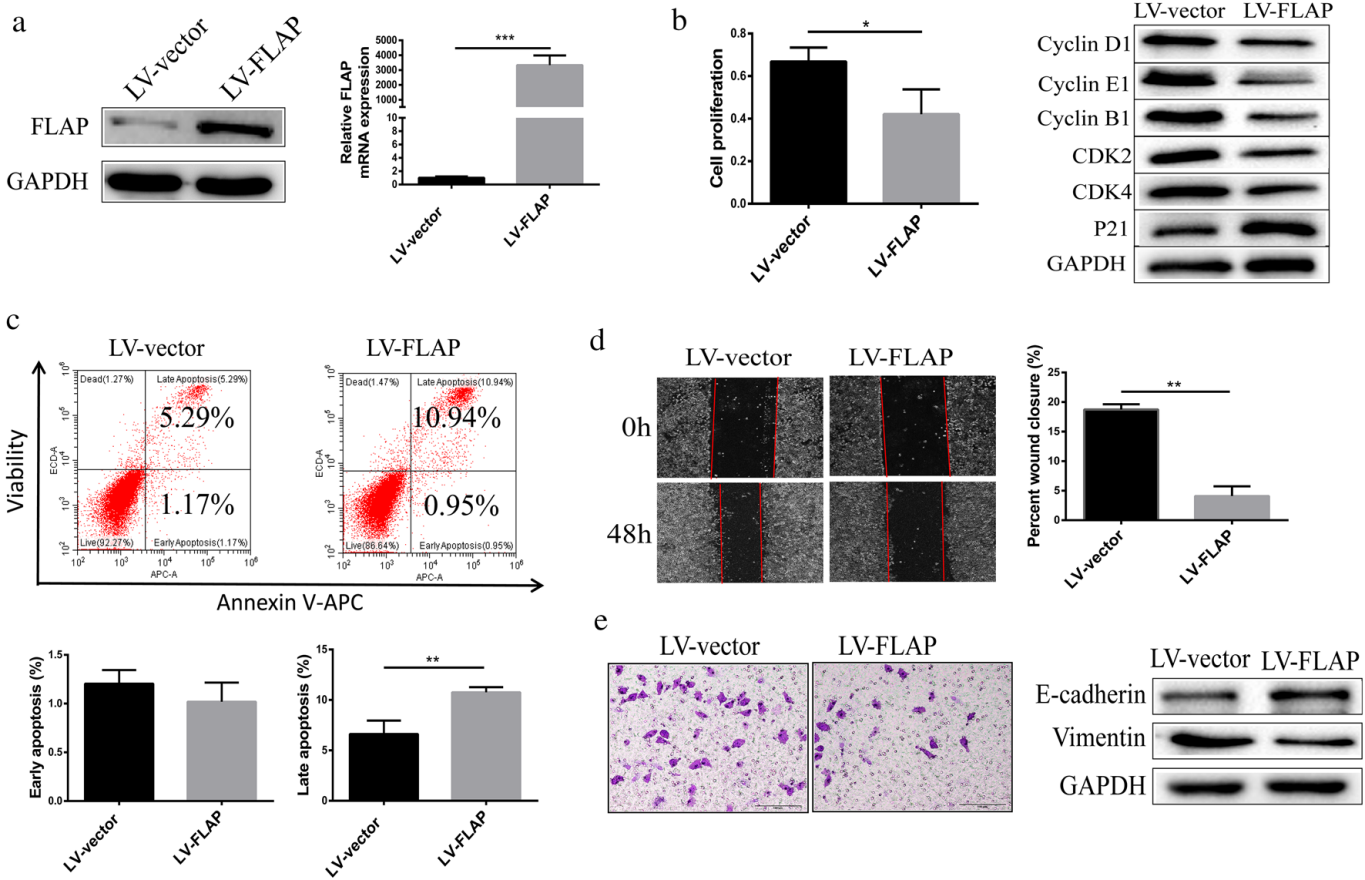
High FLAP expression inhibited HepG2 cell proliferation, cycle progression, migration and invasion. **a** FLAP protein and transcript levels of HepG2 cells transfected with control (LV-vector) or Lentivirus packaged FLAP overexpression vector (LV-FLAP). **b** Representative CCK-8 proliferation assays (left) and the cell cycle regulatory proteins expression in HepG2 cells. **c** Flow cytometry analysis (left) and quantitation (right) in HepG2 cells. **d** Representative wound-healing scratch assays (left) and quantitation (right) in HepG2 cells. **e** Representative matrigel invasion assay (left) and adhesion markers expression (right) in HepG2 cells. Data are presented as mean ± SEM. n = 3; *P < 0.05, ***P < 0.001,****P < 0.0001

Given that LV-miR-146a substantially reduced FLAP protein and mRNA expression, and miR-146a promotes HepG2 cell proliferation, it was speculated whether FLAP restrained HepG2 cell proliferation. To investigate this, HepG2 cells were stably transfected with control (LV-vector), or Lentivirus packaged FLAP overexpression vector (LV-FLAP). To investigate this, WB and qRT-PCR (Fig. 5a) confirmed that the LV-FLAP substantially elevated FLAP protein and mRNA expression. We followed proliferation for 24 h with CCK8 assays and found that the LV-FLAP significantly reduced the proliferation of HepG2 cells (Fig. 5b).

Next, we examined the expression of cell cycle regulatory proteins: Cyclin D1, Cyclin E1, Cyclin B1, p21, CDK4, and CDK2. Expression of Cyclins D1, E1, B1, CDK2, and CDK4 was efficiently decreased in cells in which FLAP was overexpressed, whereas the expression of p21 was elevated (Fig. 5b). These results suggest that a high FLAP expression reduced HepG2 cell proliferation by inducing G2/M cell cycle arrest.

Next, we evaluated the effect of LV-FLAP on cell apoptosis. Flow cytometry analysis revealed that LV-FLAP significantly increased the apoptosis of HepG2 cells (Fig. 5c).

To determine whether high FLAP expression reduced HepG2 cell migration, we performed wound-healing scratch assays in cells in which FLAP was overexpressed. At 48 h post-scratch, HepG2 cells with high FLAP expression were significantly less migratory than inhibitor NC (Fig. 5d). To evaluate whether high FLAP expression reduced HepG2 cell invasion, we performed Matrigel invasion assay in cells in which FLAP was overexpressed. Compared to LV-vector, HepG2 cells with high FLAP expression were significantly less capable to invade through Matrigel (Fig. 5e). Next, we examined the expression of adhesion markers: E-cadherin and vimentin associated with EMT. We found that cells in which FLAP was elevated had a higher E-cadherin expression and lower vimentin expression than LV-vector (Fig. 5f). Together, these results suggest that a high FLAP expression reduces HepG2 cells migration and invasion.

### MiR-146a promoted HepG2 cell proliferation, cycle progression, migration, and invasion by targeting FLAP mRNA

**Fig. 6:**
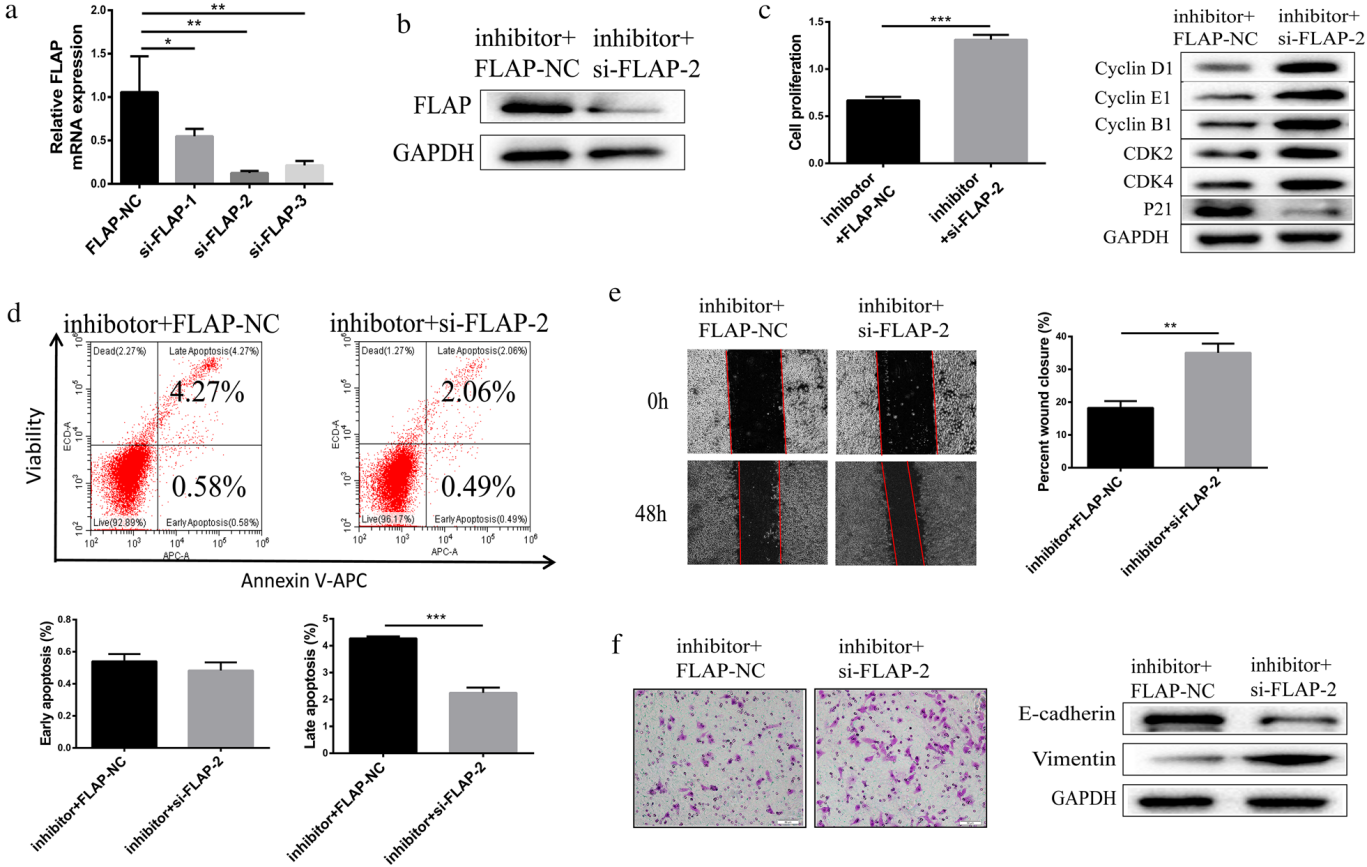
MiR-146a promoted HepG2 cell proliferation, cycle progression, migration and invasion by targeting FLAP mRNA.** a** FLAP protein and transcript levels of HepG2 cells in which stably transfected with miR-146a inhibitor transiently transfected with FLAP-NC (control) or FLAP-targeted small interfering RNA (siRNA). **b** Representative CCK-8 proliferation assays (left) and the cell cycle regulatory proteins expression in HepG2 cells. **c** Flow cytometry analysis (left) and quantitation (right) in HepG2 cells. **d** Representative wound-healing scratch assays (left) and quantitation (right) in HepG2 cells. **e** Representative matrigel invasion assay (left) and adhesion markers expression (right) in HepG2 cells. Data are presented as mean ± SEM. n = 3; **P < 0.01, ***P < 0.001,****P < 0.0001

To further confirm that miR-146a promoted HepG2 cell proliferation, cycle progression, migration, and invasion by targeting FLAP mRNA, FLAP-NC (control) or FLAP-targeted small interfering RNA (siRNA) were transiently transfected to HepG2 cells, and which were stably transfected with miR-146a inhibitor. We called them “HepG2 cells with inhibitor + FLAP-NC” and “HepG2 cells with inhibitor + si-FLAP”, respectively. QRT-PCR confirmed that FLAP-targeted small interfering RNA-2 (si-FLAP-2) had the best interference effect compared to that of the FLAP-NC group (Fig. 6a). Si-FLAP-2 was used in subsequent experiments. WB confirmed that the si-FLAP-2 substantially reduced FLAP protein expression in HepG2 cells, which were stably transfected with miR-146a inhibitor (Fig. 6b). We followed proliferation for 24 h with CCK8 assays and found that the inhibitory effect of miR-146a inhibitor on proliferation of HepG2 cells was neutralized by si-FLAP-2 (Fig. 6c).

Next, we examined the expression of the cell cycle regulatory proteins: Cyclin D1, Cyclin E1, Cyclin B1, p21, CDK4, and CDK2. Expression of Cyclins D1, E1, B1, CDK2, and CDK4 was efficiently increased in HepG2 cells with inhibitor + si-FLAP-2, whereas the expression of p21 was reduced (Fig. 6c). These results suggest that the inhibitory effect of miR-146a inhibitor on proliferation of HepG2 cells was neutralized by si-FLAP-2.

To determine whether miR-146a inhibited HepG2 cell apoptosis by targeting FLAP mRNA, we performed apoptosis assays in HepG2 cells with inhibitor + FLAP-NC and HepG2 cells with inhibitor + si-FLAP-2. Flow cytometry analysis revealed that the promoting of miR-146a inhibitor on apoptosis of HepG2 cells was neutralized by si-FLAP-2 (Fig. 6c).

To determine whether miR-146a promoted HepG2 cell migration by targeting FLAP mRNA, we performed wound-healing scratch assays in HepG2 cells with inhibitor + FLAP-NC and HepG2 cells with inhibitor + si-FLAP-2. At 48 h post-scratch, HepG2 cells with inhibitor + si-FLAP-2 were significantly more migratory than HepG2 cells with inhibitor + FLAP-NC, suggesting that the inhibitory effect of miR-146a inhibitor on migration of HepG2 cells was neutralized by si-FLAP-2 (Fig. 6e). To evaluate whether miR-146a promoted HepG2 cell invasion by targeting FLAP mRNA, we performed Matrigel invasion assay in cells in HepG2 cells with inhibitor + FLAP-NC and HepG2 cells with inhibitor + si-FLAP-2. Compared to HepG2 cells with inhibitor + FLAP-NC, HepG2 cells with inhibitor + si-FLAP-2 were significantly more capable to invade through Matrigel (Fig. 6f). Next, we examined the expression of adhesion markers: E-cadherin and vimentin associated with EMT. We found that HepG2 cells with inhibitor + si-FLAP-2 had lower E-cadherin expression and higher vimentin expression than HepG2 cells with inhibitor + FLAP-NC, suggesting that the inhibitory effect of miR-146a inhibitor on the invasion of HepG2 cells was neutralized by si-FLAP-2 (Fig. 6f). Together, these results suggest that miR-146a promoted HepG2 cell migration and invasion by targeting FLAP mRNA.

## Discussion

We present several pieces of evidence indicating that miR-146a and FLAP play an important role in HCC pathogenesis. First, we found the expression of miR-146a in HCC tissues and liver cancer cell lines was higher than that in para-carcinoma tissues and normal hepatocytes. Although there was no difference in miR-146a expression between six pairs of HCC tissues and para-carcinoma tissues, the overall expression of miR-146a was significantly higher in HCC tissues than in para-carcinoma tissues (16 HCC tissues). This is consistent with multiple studies that showed an elevated expression of miR-146a in exosomes of HCC patients [13, 29]. Moreover, expression levels of miR-146a were increased not only in HBV/HCV-infected hepatocyte and liver tissues [3, 10], but also in natural killer cells (NK) and T cells from Chronic Hepatitis B (CHB) and HCC patients [30, 31]. However, Rong et al. reported that miR-146a expression in 85 HCC tissues was lower compared to that in adjacent non-cancerous hepatic tissues [32]. Further studies should collect more samples to explore the expression of miR-146a in HCC. We also revealed for the first time that FLAP mRNA and protein were expressed at a low level in HCC tissues and HCC cell lines, compared to para-carcinoma tissues and normal hepatocytes. Previous studies have shown that FLAP mRNA was primarily expressed in various myeloid cells of the lungs and the immune system, including polymorphonuclear leukocytes (neutrophils and eosinophils), monocytes/macrophages (including foam cells of human atherosclerotic tissue), dendritic cells, mast cells, and B lymphocytes [21, 33]. Since the liver is not the primary organ for FLAP expression, reports of FLAP in the liver are uncommon. Our results are consistent with an earlier study from Kennedy BP which found that HepG2 cells received minimal activity after transfection with FLAP promoter [34], indicating that HepG2 cells do not express FLAP mRNA. We have demonstrated that an inverse relationship exists between FLAP protein and miR-146a expressions in HepG2 cell lines. Luciferase assays demonstrated the direct and specific nature of the interaction of miR-146a with FLAP mRNA. Our vitro data demonstrated that miR-146a promotes HepG2 cell proliferation, cell cycle progression, migration, and invasion, particularly by targeting FLAP mRNA. In contrast, FLAP inhibits HepG2 cell proliferation, cell cycle progression, migration, and invasion.

In our study, decreased cell viability and associated cell cycle regulatory protein expression in miR-146a-silenced cells indicates its pro-proliferative role in HCC. Many studies have shown that miR-146a promotes cancer growth. miR-146a can promote the proliferation of cervical cancer cells, suggesting that miR-146a plays a carcinogenic role in cervical cancer [35]. During HCC treatment, miR-146a increases the resistance of HCC cells to antitumor drugs [36]. However, Zu et al. revealed that miR-146a suppresses hepatocellular carcinoma by downregulating TRAF6 [37]. Our previous review also highlighted the dual role of miR-146a in the immune response and pathogenesis of HCC. Although some studies have shown that increased levels of miR-146a are associated with HCC [11, 13, 35, 36], others have revealed that it suppresses cancer cell proliferation, invasion, and metastasis [12, 38, 39]. A single miRNA can regulate multiple mRNA transcripts, and a single mRNA transcript can be regulated by multiple miRNAs. Thus, it is reasonable for miR-146a to regulate various expressions of different characteristics and functions in specific inflammatory states of HCC by downregulating various target genes. We also found that miR-146a promotes migratory and invasive behaviors of HepG2 cells by decreasing E-cadherin expression and increasing vimentin expression. Similarly, previous studies revealed that HCC exosomes were enriched with miR-146a-5p and promoted M2-polarization. Inhibiting miR-146a-5p expression in HCC reduced the expression of inhibitory receptors on T cells, reversed T cell exhaustion, and delayed HCC progression in DEN/CCL4-induced HCC mice [13]. However, Zhang et al.[12] demonstrated that miR-146a acted as a tumor suppressor in HCC metastatic cancer cells and reduced the invasion and migration of cancer cells by upregulating the expression of adenomatosis polyposis coli (APC), a tumor suppressor. Thus, the levels of miR-146a expression were associated with cancer cell metastasis in a dual role (inhibitory and stimulatory) [40].

We revealed for the first time that FLAP mRNA and protein had low levels of expression in HCC tissues and HCC cell lines, compared to para-carcinoma tissues and normal hepatocytes. In 2008, a study of liver microsomes derived from embryonic stem cells showed a steady increase in FLAP expression during the differentiation and development of mouse embryonic stem cells into hepatocellular like cells [41]. Other studies have shown that there is little to no FLAP mRNA expression detected in the adult liver, while some studies have shown that FLAP mRNA is highly expressed in the liver of the mouse embryo at day 11.5 [42], since the liver is the main hematopoietic organ of the developing mouse embryo during this period. Together, these two studies suggest that FLAP plays an important role in embryonic liver development. Our vitro data demonstrated that FLAP inhibits HepG2 cell proliferation, cell cycle progression, migration, and invasion. A previous study has shown that FLAP is universally expressed in 20 types of epithelial cancer cell lines including colon cancer, lung cancer, breast cancer, and prostate cancer [22]. Additionally, FLAP has been reported to become a prognostic predictor in several cancer types, including colorectal cancer [43], and lung adenocarcinoma [44]. Currently, drugs targeting the FLAP protein, such as BAY-X1005 and AM-103/GSK2190914, have been actively developed in humans to reduce the incidence of ischemic myocardial infarction and to treat asthma [45, 46]. Although many studies have shown that inhibition of FLAP can significantly improve ischemic myocardial infarction [45], asthma [46], arthritis [47], breast cancer [23], lung cancer [25], and other diseases, the results of this study showed that FLAP can promote apoptosis of HepG2 cells and inhibit migration and proliferation of HepG2 cells. This may be due to differences in characteristics of different tumor cells. The inhibitory effect of FLAP on proliferation, migration, and invasion of HCC HepG2 cells and the promotion of apoptosis still needs to be studied further.

Since the effects of miR-146a and FLAP on HCC cells showed opposite trends, we speculated that miR-146a might promote cancer by targeting the expression of FLAP mRNA. Luciferase assays demonstrated the direct and specific nature of the interaction of miR-146a with FLAP mRNA. To further demonstrate that miR-146a promoted HepG2 cell proliferation, cycle progression, migration, and invasion by targeting FLAP mRNA, we added the rescue experiment in which the miR-146a inhibitor stabilized HepG2 cells were further transfected with FLAP-targeted siRNA. The rescue experiment showed that the inhibitory effect of miR-146a inhibitor on proliferation, migration, and invasion of HepG2 cells was neutralized by FLAP-targeted siRNA. However, our study has some limitations. We did not conduct experiments using primary HCC cells to determine if similar results can be obtained. Additionally, we did not demonstrate the role of miR-146a and FLAP in vivo, for example in an animal model.

Recent evidence suggests that miRNA is a suitable target for the treatment of cancer, based on the long-term effects in the process of regulating multiple components of the same cellular/physiological pathway. MRX34, a liposomal formulation of miR-34a and a potential first-in-class miRNA mimic cancer therapy, has been explored in human clinical trial [48]. The results of the present study demonstrated miR-146a plays an important role in HCC progression by potentially targeting the expression of FLAP mRNA. Thus, inhibition of miR-146a expression could inhibit the development of HCC and the expression of miR-146a may serve as a prognostic biomarker and a potential therapeutic target for the treatment of HCC patients.

## Conclusion

We found that miR-146a expression was overexpressed, while FLAP protein and mRNA were suppressed in hepatocellular carcinoma tissues and HepG2 cells compared to para-carcinoma tissues and HL–7702 cells. Dual luciferase reporter gene assay showed that miR-146a-5p can directly target FLAP mRNA. Knockdown of miR-146a also resulted in increased FLAP expression of cancer cells. Additionally, miR-146a silencing or restoration of FLAP led to a reduction of HepG2 cell proliferation, cell cycle progression, migration, and invasion. This study showed that miR-146a has a stimulatory role in HepG2 cells and promotes HepG2 cell migration and invasion by targeting FLAP mRNA. Thus, miR-146a may be a tumor promoter and a potential therapeutic target for the treatment of HCC patients.

## Data Availability

The datasets generated during the current study in Fig. 2a are available in the TargetScan Release 8.0, [http://www.targetscan.org/vert_80/]. The data used in this study are included in the article.

## Abbreviations

FLAP/ALOX5AP: 5-Lipoxygenase activating protein
3'–UTR: 3'–Untranslated region
miR-146a: MicroRNA-146a
HCC: Hepatocellular carcinoma
HBV: Hepatitis B virus
HCV: Hepatitis C virus
AA: Arachidonic acid
PG: Prostaglandins
TX: Thromboxane
LT: Leukotrienes
NK: Natural killer cells
CHB: Chronic Hepatitis B
